# Editorial

**Published:** 1891-11

**Authors:** 


### Editorial.

### TARIFFS GOVERNING RAILROAD SURGEONS.

Few questions have been more agitated within the last few years than that of the mutual relations of the railroad companies and the surgeons employed by them, and at this time the question is under consideration of the American Medical Association for report at the next meeting.

But as we understand the matter, that body will deal more with the ethical aspect of the contract, and the right of a surgeon to enter into such an agreement, and it is not our purpose at this writing to consider the merits of this view of the case, but to call attention to the rates of charges that are being adopted by some of the companies. From somewhat intimate association with the officers of railroad companies, and a study of the question from its various aspects, we are convinced that there are two sides to the matter, and there are extremists on each of them.

The arguments advanced from the professional side are that the surgical service rendered is worth as much as it would be in any other case, and the companies are able to pay, so that the charge should be for a full fee, based upon the fact of the individual being fully able to pay. That because the work is paid for by a corporation, it should not be charged for at a lower rate than in the ordinary rounds of a private practice. We admit that there is much in this, but we should also hear the other side.

The managers of railroad companies, in determining the rate fixed for compensation, say that, in the first place, the company places in the hands of the surgeon its entire work; that in a very large majority of instances the service is of a charitable nature on their part, and independent of any legal liability; that it occurs among a class of men who are unable to pay full fees for medical attention, and should the account be paid, it would be only in small payments, but probably not at all; that in the case of their surgeons, the bills are paid promptly, and in bulk. As put by the general manager of a large company in conversation with the writer, "It is wholesale and sure pay, against retail and slow pay."

Looking at the matter from both sides, we are convinced that in the fixing of rates the companies have a clear right to expect a leniency on the part of their surgeons, and at the same time they should not go to the other extreme, and fix rates under which no respectable surgeon can consent to work. As a rule the tendency has been to somewhat increase the charges within the last two years, until now the fees that are allowed by most companies are low, but under the circumstances fair.

One exception to this statement must be made in reference to a "tariff" which came into our hands a short time since, which is somewhat a curiosity. We refer to that governing the surgeons of the East Tennessee, Virginia and Georgia Railway, one of whose important stations is the city of Atlanta. We are only able to make a few extracts from the schedule, in order to give some idea of the general tendency of the whole, and the value that is attached to the services of its surgeons.

By this tariff all of the minor operations in the way of dressing contusions and compound fractures of the fingers, and the many hand and foot injuries, the surgeon is paid for the first visit $3.00, and is required to furnish his own dressings. This class also includes all burns and scalds, independent of size and extent. One dollar for subsequent visits, dressings furnished. In the list of amputations we find leg and forearm $30.00, respectively, and arm, thigh, shoulder and hip joint, $40.00. For dislocations, from $5.00, to $10.00 is paid for all joints except the hip, which is $40.00. In fractures of the smaller bones, including the ribs, $5.00 is allowed for the dressing, and $1.00 for subsequent visits. All other fractures, including the thigh, are rated at $10.00 for the first dressing, the surgeon to pay for the splints and dressing used. After the first dressing, all visits are to be charged at the uniform rate of $1.00, with no extra allowance for night visits, or for detention. In the case of the surgeon being called from the city, he is permitted to make an additional charge of ten cents per mile mileage one way, though no fee for detention. Some idea may be formed as to the results when we figure that the surgeon might be called, for instance, to Dallas, Ga., which would take all of one day, by the company's schedule of trains, and for this visit, and the dressing of a fractured thigh, he would be allowed the colossal charge of $13.40, out of which he must pay for the dressing! Thus the charges run through the list, of which these are samples, and only selected to illustrate the whole.

In the face of these figures one feels dismayed, and two thoughts are suggested: First, how could the chief surgeon of any company have the temerity to submit them to a staff of surgeons; and second, how he can find any reputable medical man who would consent to follow them. One would think that it would be the policy of a railroad company to secure competent surgeons, for upon their work will, in many cases, the measure of money paid out for injuries be determined, but surely they could not, for the pittance here shown, expect good results. When the surgical field is occupied by gentlemen who do not value their services any more highly than this, those who try to follow the art as it must be to be successful, can but feel that the prospect is highly discouraging. It can be readily seen that the dressings alone in many cases, if properly applied, would cost more than the company allows in the way of a fee.

After all, it is a fact in medicine, as well as other callings, that one's time and talent commands what it is worth, and in this case we are sure that there will be no exception to the rule. The personnel of the staff will be commensurate with the compensation offered. In the end the railroads will find that cheap surgery is a very expensive investment. The offer of such terms is in the nature of an affront to any reputable surgeon.

### THE DOCTOR BILL VETOED.

In our last issue we gave some few remarks as expressing our views in regard to the doctor bill recently passed by the State Legislature. At that time our remarks were in condemnation, while our present utterances will go to the other extreme—in commendation. This radical change has been effected by our level-headed Governor, who, in his wisdom, has seen fit to veto the bill. The last legislature was sadly in need of a check to their voluminous production of bills, the majority of which were introduced by those who felt a necessity of "doing something." The amount of money which will be expended by their enactments is simply fabulous. It is with pleasure that we can anticipate the favorable criticisms from our journalistic colleagues upon this action of our chief magistrate, who, by one stroke of the pen, has shown to the world that to physicians are due all the rights and privileges of his fellow-men. While we are often able to bear an insult through our charity at the ignorance of the giver, we well knew that our Governor would be guilty of no such association. All fair-minded people will commend his action in vetoing the bill.

---

DR. MARK O'DANIEL, who for years was one of the assistant physicians of Georgia lunatic asylum, is now in New York, attending the New York Polyclinic. He is taking a special course in nervous diseases, which he expects to make a specialty. He is going to locate in Macon, Ga. The people of that city are to be congratulated to have such a doctor and gentleman to attend to their ills. Mark, we wish you great success in your new home.

---

H. M. WHELPLEY, Ph. G., M. D., F. R. M. S., now occupies the chair of Physiology and Histology in the Missouri Medical College. He is also secretary of the faculty and director in Histological Laboratory. The doctor is professor of microscopy in the St. Louis college of pharmacy, and edits *Meyer Brothers' Druggist.*

---

THE PREVENTION OF RUST ON SURGICAL NEEDLES.—Dawbarn (*New York Medical Journal*, September, 1891,) advises the following procedure for this purpose: The needles are to be immersed in benzine to remove grease and then run through a towel. A piece of cork, the size of a pea, is placed on the point of each needle, and they are then kept in a wide-mouthed glass-stoppered bottle filled with absolute alcohol. After using, the needles are to be passed repeatedly through a thick soapy towel, the eye cleansed with a thread, and then placed in benzine and afterward kept in absolute alcohol.—Ex.

### THE DIAGNOSIS OF HEAD INJURY FROM DRUNKENNESS.

The subject brought forward by Mr. Battle in The Lancet of Sept. 12th, 1891, is one of the first importance, especially to the surgeons of police who have to treat the patients. As surgeon to the C division of the Metropolitan Police, this subject in one form or another, has been frequently brought to my attention, and some years since I wrote a brochure with the hope of assisting my brother surgeons in forming an opinion on, and in the treating, these cases he describes. Unfortunately, they are only a small portion of those which may be roughly classified as due to pressure on the brain. The diagnosis would be sufficiently difficult were these straightforward cases ; but unfortunately, with a few exceptions, they have a halo of alcohol surrounding them, masking or intensifying the symptoms upon which we rely to form our opinion. Of course, a police station, or cell, is not a proper place to make an elaborate diagnosis, and our instructions and desires are to have such cases removed to a hospital ; but, unless the symptoms are pronounced, the hospital surgeons cannot take them in, and the surgeons at the workhouse infirmaries, if they have any doubt, will not do so. We have thus a responsibility thrown upon us most undesirable and perplexing. If we remove the element of drink which, as I have said, hides the symptoms proper to brain trouble, we are more likely to be of service to the patient and to relieve ourselves of this great responsibility. Feeling this want, as all must who are brought into contact with these cases, I have for some years employed ammonia as more generally applicable than any other means of treatment. The preparation I use is the iquor ammoniae fortior, which I allow insensible patients gurdedly to inhale until they are sensible to some extent of irritating action ; when the patients are able to swallow, three drops of the ammonia in a tablespoon of water are put far back in the mouth. The beneficial effects are soon seen ; the fumes of drink vanish, and the symptoms proper to the particular brain mischief, if any, stand out more clearly. It is seldom necessary to continue the administrations, though I have occasionally done so, and I have never seen any bad effects follow, as I at first thought possible.—*J. H. Waters in the Lancet.—Nashville Jour. Med. and Surg.*

### A BIRTH OF A LIVE CHILD AT SIX MONTHS AND A HALF.

DR. H. COLLYER IN N. Y. MEDICAL JOURNAL.

Reported a case of this nature. The woman was thirty-three years of age, had been married eleven years, and had had four children at term and one miscarriage. She had been under the speaker's care for chronic pelvic peritonitis, which fact had given him opportunities for observation in the case that he might not otherwise have had. On July 5, 1890, she menstruated as usual. On August 6th there was a scanty flow for three days. During the third week in August changes were noticed in the uterus characteristic of the earlier weeks of pregnancy. The woman stated that conception must have occurred on July 13th, denying its possibility before that date. On January 4, 1891, while she was working a sewing machine, there was a sudden and profuse hemorrhage All attempts to prevent labor were futile, and on February 3d she was delivered of a small male child weighing two pounds and two ounces. The child cried at once, and, wrapping it up warmly, the speaker waited for some five minutes till pulsation in the cord had ceased before separating the child from its mother. The placenta gave some trouble, and there was found attached to it an independent lobule. The bones of the child's skull were soft and overlapping. The testes had not descended, and the finger nails were only just showing. The infant was wrapped in wool and put in a basket, which was placed near a fire kept continually burning. At first the child was fed with milk and water from a spoon every two hours, and subsequently from the breast. It has since continued to thrive in every respect. The date at which the child was born and the general condition of its development would indicate the period of gestation at six months and a half.

